# Molecular Targets for Components of Essential Oils in the Insect Nervous System—A Review

**DOI:** 10.3390/molecules23010034

**Published:** 2017-12-23

**Authors:** Milena Jankowska, Justyna Rogalska, Joanna Wyszkowska, Maria Stankiewicz

**Affiliations:** 1 Department of Biophysics, Faculty of Biology and Environmental Protection, Nicolaus Copernicus University, Toruń, Poland; Lwowska 1, 87-100 Toruń, Poland; jwyszk@umk.pl (J.W.); stankiew@umk.pl (M.S.); 2 Department of Animal Physiology, Faculty of Biology and Environmental Protection, Nicolaus Copernicus University, Toruń, Poland; Lwowska 1, 87-100 Toruń, Poland; rogal@umk.pl

**Keywords:** acetylcholinesterase, bioinsecticides, essential oils, GABA receptors, insect nervous system, octopamine receptor

## Abstract

Essential oils (EOs) are lipophilic secondary metabolites obtained from plants; terpenoids represent the main components of them. A lot of studies showed neurotoxic actions of EOs. In insects, they cause paralysis followed by death. This feature let us consider components of EOs as potential bioinsecticides. The inhibition of acetylcholinesterase (AChE) is the one of the most investigated mechanisms of action in EOs. However, EOs are rather weak inhibitors of AChE. Another proposed mechanism of EO action is a positive allosteric modulation of GABA receptors (GABArs). There are several papers that prove the potentiation of GABA effect on mammalian receptors induced by EOs. In contrast, there is lack of any data concerning the binding of EO components in insects GABArs. In insects, EOs act also via the octopaminergic system. Available data show that EOs can increase the level of both cAMP and calcium in nervous cells. Moreover, some EO components compete with octopamine in binding to its receptor. Electrophysiological experiments performed on *Periplaneta americana* have shown similarity in the action of EO components and octopamine. This suggests that EOs can modify neuron activity by octopamine receptors. A multitude of potential targets in the insect nervous system makes EO components interesting candidates for bio-insecticides.

## 1. Introduction

Essential oils (EOs) are natural, complex substances extracted from different plant organs, and terpenoids are the main components of them [1]. People have taken advantage of EOs as well as their particular components for many centuries. Recently, the historical aspects of use of EOs has been described in detail [2,3]. Nowadays, we know more than 3000 kinds of EOs, about 300 of which are currently used. In traditional agriculture, farmers apply EOs to protect stored grain. EOs are widely utilized as insect repellents, mainly against mosquitoes [3,4,5,6]. EOs obtained from lemon and eucalyptus are used as the active substances in non-toxic repellent products that are recommended for children. Moreover, several studies demonstrate that EOs do not only repel the insects but also act on them as neurotoxic compounds [6,7,8,9,10,11,12,13,14,15,16,17,18].

It has been proved that EOs from 1500 plant species have insecticidal properties and are efficacious regarding both forms of insects—adults and larvae. For example, eugenol is toxic to a number of insect orders: Coleoptera, Hymenoptera, Isoptera; citral to insect species: *Ceratitis capitata* and *Anastrepha fraterculus*; geraniol to *Aedes aegypti*, *Aedes albopictus*, *Anopheles quadrimaculatus*, thymol to *Culex tritaeniorhynchus*, *Aedes albopictus* and *Anopheles subpictus*. All of them are also toxic to the cockroach *Periplaneta americana* [19,20,21,22,23,24,25,26,27,28]. Additionally, extensive research has provided evidence that some EO components, applied in binary mixture, can exhibit synergistic or antagonistic activity. Such effects suggest diverse mechanisms of action of EO components [29,30,31].

Eugenol, α-terpineol and L-carvol cause hyperactivity in insects at first. Stretching the legs and numbness precede the insect’s death [19,20,21,22,23,24,25,26,27,28]. These effects demonstrate the neurotoxic activity of EOs and motivate the investigation of their molecular targets in insect organisms. Understanding the spectrum of action of EOs on insect targets could be crucial for the application of EOs in the development of new, natural insecticides. The aim of this article is to present some identified targets for EOs and shed some light on their mode of action.

## 2. Essential Oils—Inhibitors of Acetylcholinesterase

**Figure 1:**
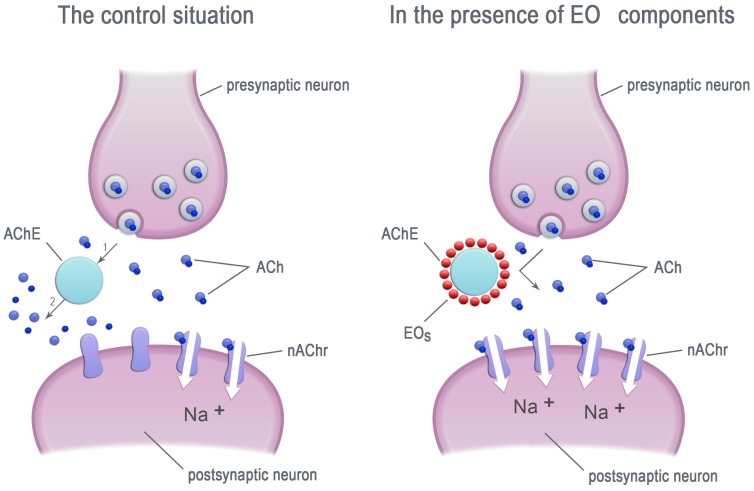
The EO components inhibit the acetylcholinesterase (AChE) activity. ACh—acetylcholinesterase, nAChr—nicotinic acetylcholine receptors, EOs—essential oil components.

A lot of research demonstrates that EOs inhibit the activity of acetylcholinesterase (AChE) (Figure 1) (e.g., [32]), which is one of the most important enzymes in neuro-neuronal and neuromuscular junctions in both insects and mammals [33,34,35]. Since the insect AChE differs from the mammalian one by a single residue, known as the insect-specific cysteine residue, AChE can be an insect-selective target for the newly developed insecticides, safe for non-target vertebrates [36,37,38,39,40]. Essential oils are estimated to be a potential source of insecticides due to their ability to modifying the insect AChE activity [41,42,43,44,45,46,47,48,49,50,51].

It has been demonstrated that EOs from the following plants can inhibit AChE: *Chamaemelum nobile*, *Eriocephalus punctulatus*, *Ormenis multicaulis*, *Santolina chamaecyparissus*, *Cyclotrichium niveum*, *Thymus praecox* subsp. caucasicus var. caucasicus, *Echinacea purpurea*, *Echinacea pallida*, *Salvia chionantha*, *Anethum graveolens*, *Salvia lavendulaefolia* [41,42,43,44,45]. Moreover, the properties of the isolated components of EOs have been examined as well (Table 1, Table A1—Appendix A). Forty-eight of 73 examined substances exhibited an anti-AChE activity. However, the experiments were mainly conducted on the isolated AChE from the electric eel (*Electrophorus electricus*) and from some species of mammals. Only 28 components were tested on insect AChE and 23 of them inhibited the enzyme. The most efficacious of them were: α-pinene and β-pinene, β-phellandrene, carvacrol, limonene, menthol, menthone, 1,8-cineole, *cis*-ocimene, niloticin [41,47,48,49,50,51,52,53,54]. Most of the EO components displayed anti-AChE activity in mM concentration. Only one study proved the inhibitory effect of EOs component (carvacrol) on AChE in μM concentration [48].

To understand the effectiveness of EOs in AChE inhibition we need to consider their exact mode of action and also to recognize the type of inhibition. First of all, knowledge concerning the modification of enzyme kinetics is necessary. In the majority of papers there is no data related to the changes in the AChE kinetics after the EOs’ administration. The available research shows that some of the EO constituents function as competitive inhibitors and others as uncompetitive inhibitors (Table 2) [55,56,57,58,59,60]. It is also difficult to explain the EOs’ mode of action because the activity of EOs as complex compounds differs from the activity of their single components. For example, EO from the tea tree (*Melaleuca alternifolia*) is an uncompetitive inhibitor, while its particular components are competitive inhibitors [57]. Such competitive inhibitors attach to the active sites in AChE and prevent the binding of ACh. It causes the decrease in the binding of the neurotransmitter but the maximal activity of the enzyme remains unchanged. On the other hand, the uncompetitive inhibitors bind to other sites of AChE and allosterically alter the action of the enzyme. They bind rather to the enzyme-substrate complex than to the enzyme itself and thus prevent product formation. As a result, the maximum activity of the enzyme decreases. Therefore, different inhibitory action of EOs on the AChE suggests the existence of diverse binding sites in the enzyme molecule.

The AChE enzyme has a deep “active-site gorge” with two target sites: “catalytic” at the bottom and “peripheral” at the entrance [61]. “Dual binding site” inhibitors interact with the AChE at both the catalytic and the peripheral site [62]. Thus, they can act both as competitive and uncompetitive inhibitors. EO components can act as dual inhibitors if they form a blend. López et al. [60] analyzed the kinetics of the inhibition and the spatial size of terpenoids on their binding capability. They conclude that two monoterpenoids can bind to one AChE molecule at a time. The binding of the first EO component favors the attachment of the second one. Moreover, by using a molecular docking, they demonstrated that some components (carvone and fenchone) can bind to several binding sites in the AChE. In contrast, they found only one binding site for terpinene and camphor.

The data described above may suggest a synergistic action of the EO components. In fact, the majority of essential oils exhibit greater activity than their single components. However, we have found only a few papers where the synergistic action of the EO components was estimated using statistical analysis of interaction. Savalev et al. [63] have proposed synergism between 1,8-cineole and α-pinene. They obtained similar results for 1,8-cineole and caryophyllene oxide. On the other hand, an antagonistic interaction was found between 1,8-cineole and camphor. Miyazawa et al. [64] also observed antagonism between some EO components. They compared the inhibitory effect of the natural EOs extracted from the plant with the sum of the inhibitory effectiveness of the major single components and with the “artificial” mixture of them. EOs exhibited the highest inhibition (46%), the sum of the inhibitory action of the EO components was lower (29.5%) and the “artificial” mixture of the EO components inhibited the AChE only by 19%. On the other hand, the study by Jukic et al. [65] showed that thyme EO exhibited less activity than its single components. Certainly, the positive or the negative interaction between the EO components depends on their relative quantity.

The structure-activity relationships for EOs are also unclear. It is difficult to define which chemical type of EO compounds is more active. Lee et al. [50] have suggested that monoterpenoid ketones are more active than alcohols or aldehydes. However, among 6 inactive compounds, two were ketones. In the same study, menthone (ketone) has a lower inhibitory activity than others. Moreover, among active compounds, two were phenolic alcohols. Certainly, it would be necessary to identify other features of chemical structure (e.g., double bond in phenolic ring) to determine the activity of the EO constituents. López et al. [60] have found a correlation between the size of the tested components and their inhibitory activity on the AChE. The substance with higher spatial size exhibited higher activity. Reegan et al. [53] performed a molecular docking of niloticin (large spatial size terpenoid) to the AChE of *Aedes aegypti*. They showed a high binding affinity of niloticin to the AChE and determined the binding residues as THR’58 and HIS’62. However, Dambolena et al. [66] provided mathematical analysis of factors affecting insecticidal activity of EO components and they discovered that compounds with lower molar volume and fewer rings are more active.

To sum up, the study on EOs as AChE inhibitors showed that monoterpenoids appeared to be weak AChE inhibitors. The inhibition of AChE requires mM concentrations of EOs [48] while usually neurotoxic symptoms of EOs are visible at their concentrations smaller by 3 orders of magnitude. Additionally, the inhibition of the AChE is always fast reversible [67,68]. Moreover, the chemicals (e.g., carvone) in one study caused the inhibition of AChE but in another study, using the same AChE, no activity was shown, so the results are not reproducible. Thus, AChE inhibition does not seem to be the primary neurotoxic action of EOs, however, some of the large sized EO chemicals can be consider as AChE inhibitors.

## 3. Essential Oils—Modifiers of GABA Receptors

### 3.1. Mammalian GABAA Receptors

Gamma-amminobutyric acid (GABA) is the major inhibitory neurotransmitter in the nervous system and the muscles in both mammals and insects (however in some cases it can play a role of excitatory neurotransmitter). It binds to specific receptors (GABArs) in synaptic or extrasynaptic membranes [69,70,71,72]. In mammals there are two types of GABA receptors: ionotropic (GABA_A_rs) and metabotropic (GABA_B_rs) [73,74].

**Figure 2:**
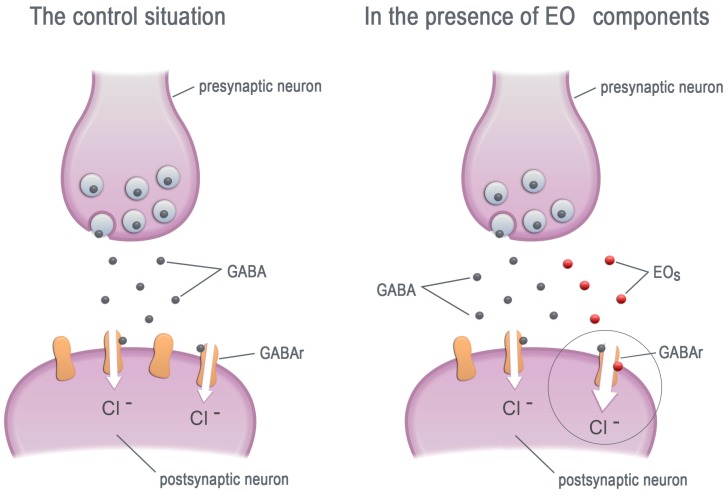
The EO components increase the chloride current by allosteric modulation of the GABA receptors. GABA—γ-aminobutyric acid, GABAr—GABA receptors, EOs—essential oil components.

Many papers report essential oils action on the GABArs, primarily belonging to the ionotropic receptor group [75]. Studies that proved the influence of essential oils on the GABArs were conducted mainly on mammals. According to a great deal of data, EOs and their components are mostly positive modulators of the GABA_A_ receptors (Figure 2). Menthol, thymol and other components increase the Cl^−^ current induced by the GABA neurotransmitter (Table 3) [76,77,78,79,80,81,82]. Such a situation occurs in low (μM) concentrations of EOs. Additionally, some of the EO constituents induce a weak Cl^−^ current themselves when applied at a concentration near 1 mM [75,76,78,80]. Higher concentrations of previously mentioned EOs do not exert any effects on GABA_A_rs probably because of the desensitization of the receptors [82]. There are also EO components that do not induce any effect on the GABA_A_rs Cl^−^ current, for example: camphor, carvone, menthon [76], linalool and α-terpineol [83].

The effect of the EO components on the GABA receptors depends on their chemical structure. Different EO stereoisomers vary in their potency to modulate the GABA receptors: (+)-menthol and (+)-borneol have higher activity than (−)-menthol and (−)-borneol [83,84]. The presence of a functional group is important as well. Alcohols have a stronger modulatory effect on the GABA_A_rs (e.g., thymol, menthol, borneol)—than ketones—(linalool, α-terpineol) [76,83].

Many studies have been performed to define the binding sites for the EO components in the GABArs [76,78,85]. However, such experiments are rather difficult to carry out in natural, neuronal membranes, because EOs are lipophilic substances and they can nonspecifically affect cellular membranes: they can increase the membrane permeability or cause damage [86]. The majority of data concerning EOs binding to the GABA_A_rs was obtained using competitive studies with already known GABA_A_rs ligands. Such experiments can only provide indirect evidences for the existence of binding sites for the EO components in the GABArs and should be complemented by more direct methods. Recent knowledge concerning the EO binding sites in the GABArs is presented below.

Although EOs do not compete with the GABA site antagonists [80], in mM concentrations EOs can cause weak Cl^−^ currents inhibited by bicuculline (a competitive antagonist of the GABA_A_rs) [84].

The EO components do not bind to the benzodiazepine site despite the fact that the action of EOs is similar to the action of the benzodiazepines. Watt et al. [78] and Granger et al. [84] have shown that flumazenil (a benzodiazepine site antagonist) did not eliminate the potentiation of a Cl^−^ current induced by menthol and borneol. However, Sánchez-Borzone et al. [87] have observed that carvone can allosterically modify the flunitrazepam binding to the benzodiazepine site.

Moreover, the EO components do not bind to the picrotoxin site. If they did bind to the picrotoxin site, they would have induced the inhibition of the GABA-induced current—but such an effect was not observed. Additionally, picrotoxin completely inhibits the GABA-induced currents modulated by borneol [80,84]. EOs are also not competitive for the radioligand [^3^H]-TBOB (non-competitive channel blocker), in contrast to all ligands of the picrotoxin site [83].

It was proposed that EOs bind to the GABA_A_rs anesthetic site. The EO components (e.g., menthol, borneol or geraniol) are structurally similar to a known ligand of the anesthetic site—propofol. Both propofol and menthol are cyclic molecules containing the hydroxyl group. Borneol and geraniol have a similar structure to propofol as well. Moreover, the action of EOs and propofol is similar—they potentiate the GABA induced Cl^−^ current. Propofol itself (in μM concentration) can also cause currents via the GABA_A_rs. In contrast, such a current was observed only after much higher (mM) concentrations of menthol. However, menthol competed with propofol and significantly decreased the propofol-induced current [78]. It was proposed that propofol binds to the GABA_A_rs between β+-β− and β+-α− subunits [88]. Additionally, propofol can bind to another β subunits combination but only at a 10× higher concentration [89]. Amino acid residues crucial for the propofol binding are located in positions: 265, 236, 296, 286, 444. These residues are also proposed as amino-acid residues participating in EOs binding [78,88,89,90].

To summarise, the EO components most probably share a binding site with propofol in the GABArs of mammals. A similar action of these compounds was proved in behavioral experiments. Both EOs and propofol cause sedation of a mouse (*Mus musculus*) [91] and a silver catfish (*Rhamdia quelen*) [92]. 

### 3.2. Insect GABA Receptors

The insect GABArs are related to vertebrate ionotropic GABArs. Similarly to vertebrates, in the insect nervous system GABArs mainly mediate the inhibitory effect on neurotransmission. However, there are several structural and pharmacological differences between the mammalian and the insect GABArs and thus the insect GABArs can be a very promising target for the development of new insecticides. The insect GABArs display features of both mammalian GABA_A_ and GABA_C_ receptors. The level of similarity between the insect and the mammalian GABArs is the same as between the insect GABArs and the insect nicotinic receptors. The insect GABArs are similar (85–99%) in different orders of insects. Three kinds of subunits were identified in the insect GABArs: RDL (resistant to dieldrin), GRD (GABA and glycine-like receptor of *Drosophila*) and LCCH3 (ligand gated chloride channel homologue 3). Among the insect GABArs subunits LCCH3 is the most similar to the mammalian GABArs—precisely to the β3 subunit of the GABA_A_rs. The resemblance in the amino acid sequence between the LCCH3 and the GABA_A_ β3 subunit amounts to 50% [93]. However, the presence of the LCCH3 subunit in insects is time- and tissue-limited. Experiments on *Drosophila melanogaster* showed that LCCH3 is located in cell bodies of the embryonic nerve cord and brain, in neuronal cell bodies surrounding the adult brain and in the olfactory system [94,95]. In contrast to the mammalian GABA_A_rs, the majority of the insect GABArs is insensitive to bicuculline and, differently than the subclass GABA_C_rs, they can be allostericaly modified by benzodiazepines and barbiturates [69].

Homomeric GABA receptors composed of the RDL subunits are accepted as a model to study the physiology and pharmacology of the insect GABArs because they are blocked by picrotoxin and they are insensitive to bicuculline [96,97]. The insect GABArs are targets for several chemical insecticides such as dieldrin, fipronil, insane, BIDN (bicyclic dinitrile convulsant). All of them act as antagonists of the GABArs and induce inhibition or overexcitation of the insect nervous system [96,97,98,99,100].

The efficacy of essential oils as insecticides was presented in many publications [101,102,103] although, the data concerning the effects of EOs on the insect GABArs are very limited. The research on RDL receptors has shown that thymol caused strong potentiation of the Cl^−^ current evoked by GABA. Moreover, thymol alone can induce a small current as well [75]. In addition, thymol, carvacrol and pulegone enhanced the binding of [^3^H]-TBOB to membranes of the insect’s neuronal cells. These monoterpenoids also increased the GABA-induced Cl^−^ uptake in the insect membrane preparations. It was proposed that these EO components are positive allosteric modulators of the insect GABA receptors [83]. It is supported by research by Waliwitiya et al. [104], who observed thymol induced reduction of flight muscle frequency at *Phaenicia sericata*, which was comparable to GABA effect. Anyway, EO action on the insect GABArs needs further studies.

## 4. Essential Oils—Ligands of Octopamine Receptors

Octopamine (OA) is an invertebrate multifunctional molecule, structurally and physiologically related to vertebrates noradrenaline. It has been found that it can act as a neurotransmitter, as a neurohormone and as a neuromodulator [105,106,107]. OA is present in the nervous system, neuroendocrine cells and hemolymph [108]. It is involved in the regulation of different forms of insect activity e.g., arousal level. It also plays an essential role in the insect stress response, aggressive behavior and social behavior [109,110,111]. Modern molecular biology techniques have made it possible to follow in detail the role of OA in the insect organism. OA binds to specific G protein-coupled membrane receptors (OAr). The binding of OA to these receptors leads (via G protein) to the activation of the enzyme adenylyl cyclase. It transforms ATP to cAMP and causes an increase in the cAMP level, which is a signaling molecule, activating the protein kinase A (PKA). G protein also activates phospholipase C. It leads to the release of calcium from deposits in the endoplasmic reticulum and to the elevation of its intracellular level as well as to the activation of the calcium-dependent protein kinase C (PKC). Protein kinases phosphorylate a number of enzymes and receptors, which, lead to the modulation of their activity. This results in important changes in cell functions [112].

Three subclasses of OAr have been distinguished—depending on the kind of the G protein-coupled. Moreover, there are two kinds of receptors for which tyramine (TA—a precursor of OA) is a ligand [113]:

- α-adrenergic-like—the binding of OA to these receptors increases the level of the intracellular calcium; the secondary effect is an increase of the cAMP level;
- β-adrenergic-like—the binding of OA to these receptors increases the level of cAMP;
- octopamine/tyramine—the receptors are similar to α2—an adrenergic receptor in mammals. It is sensitive both to OA and TA. TA binding to this receptor causes a decrease in the cAMP level. In contrast, OA binding to the receptor causes an increase in the cAMP level;
- two classes of receptors for TA only: the activation of TyrR II causes an increase of the intracellular calcium level, the activation of TyrR III induces the increase of the calcium level and the decrease of the cAMP level [114,115,116].

**Figure 3:**
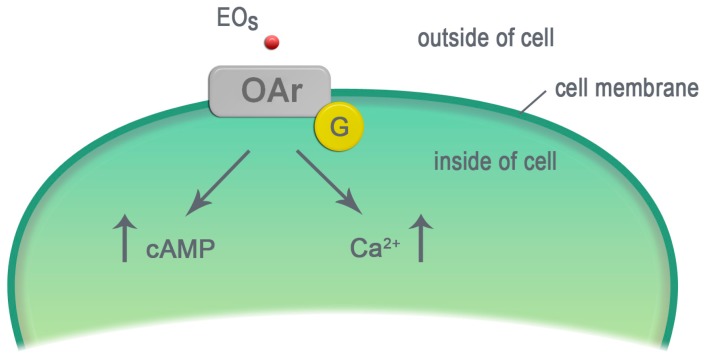
The EO components activate the octopamine receptors. EOs—essential oil components, OAr—octopamine receptor, G—protein G, cAMP—cyclic adenosine monophosphate, Ca^2+^—calcium ions, ↑—increase in the molecule level.

In several papers the authors have demonstrated that EOs act in a similar way to OA (Figure 3). Eugenol, α-terpineol and their mixture with cinnamyl alcohol induced an increase in the cAMP level. However, at higher concentrations geraniol and citral decreased the cAMP level. The same EOs reduced the binding of [^3^H]-OA to receptors [117]. Interestingly, cinnamic alcohol itself increased OA level over 20 times in *Blatella germanica* [118]. Price and Berry [28] have examined the effect of EOs on the bioelectrical activity of the cockroach (*Periplaneta americana*) ventral nerve cord and the functions of DUM neurons (dorsal unpaired median neurons) in the terminal abdominal ganglion. Geraniol and citral at low concentrations (μM) increased the spontaneous firing rate in the DUM neurons and in the nerve cord. Similar effects were observed after the OA application. However, in higher concentrations (mM) these compounds decreased the activity of the DUM neurons and the nerve cord as well. Eugenol reduced the activity of the DUM neurons and the nerve cord. The depressive effects of high concentrations of EOs may be explained by the destructive influence of EOs on neuronal membranes. A study performed by Enan [117] demonstrated that eugenol, cinnamyl alcohol, 2-phenethyl propionate and *trans*-anethole exert their toxic effects via OArs. EO components (eugenol, *trans*-anethole and 2-phenethyl propionate) increased Ca^2+^ concentrations in HEK-293 cells expressing OArs from cockroach *P. americana* and *D. melanogaster*. However, *trans*-anethole increased and eugenol decreased the cAMP level in these cells. All three of these EO components significantly decreased the binding of [^3^H]-yohimbine (ligand of OArs). Kostyukovsky et al. [119] have shown that the EO component SEM-76 caused an increase in the cAMP level, in a similar way to OA. In addition, phentolamine (OArs antagonist) abolished SEM-76-induced changes in the concentration of cAMP.

The effect of EOs was also tested on *P. americana* tyramine receptors (TArs). Thymol, carvacrol and terpineol inhibited the binding of [^3^H]-TA to membranes of S2 cells expressing TArs. Moreover, these EOs changed the cAMP level in S2 cells. The effects of EOs were observed in the μM concentrations corresponding to the physiological ligands activity [120].

All the presented data provides convincing arguments that the EO components interact with OA and TA receptors. They act mainly as agonists of these receptors. Importantly, EOs can be considered as agonists of all types of OArs and TArs. They cause an increase in both the cAMP level and in the intracellular Ca^2+^ level. Thus, they can induce the activation of kinases PKA and PKC and phosphorylation of many proteins (including ion channels, enzymes and receptors) [121]. The presence of OA in mammals is minor and no OArs was found in mammals (nevertheless, it should be taken into account that OA is prohibited in sport owing to its stimulating properties) [112]. The effects of essential oils components on octopamine receptors specific to insects lead to the conclusion that essential oils represent a very interesting source of molecules for designing the insect pest control.

## 5. Conclusions

Studies of neurotoxic effects of essential oils allowed their molecular targets to be determined: acetylcholinesterase enzymes, ionotropic GABA receptors and metabotropic octopamine receptors. The most evident proof concerns the effect of EOs on octopamine receptors, which are specific for invertebrates including insects. This fact strongly motivates future studies on EOs as bioinsecticides.

## Figures and Tables

**Table 1:** The effects of the essential oil components on the acetylcholinesterase activity in insects.

No.	Essential Oils Components	AChE Source	IC_50_ (mM)	Ki (mM)	Reference
1	Anisaldehyde	BxACE-1 from *Bursaphelenchus xylophilus*	4.95		[46]
		BxACE-2 from *Bursaphelenchus xylophilus*	8.53		[46]
		BxACE-3 from *Bursaphelenchus xylophilus*	>50		[46]
2	Camphene	*Blatella germanica*	N.A.		[47]
3	Camphor	*Blatella germanica*	N.A.		[47]
4	3-Carene	BxACE-1 from *Bursaphelenchus xylophilus*	0.37		[46]
		BxACE-2 from *Bursaphelenchus xylophilus*	8.18		[46]
		BxACE-3 from *Bursaphelenchus xylophilus*	>50		[46]
5	Carvacrol	*Musca domestica*	0.0012		[48]
		*Dermacentor variabilis*	0.0018		[48]
		*Periplaneta americana*	0.0004		[48]
		*Aedes aegypti*	0.0012		[48]
		*Drosophila suzukii*	N.A.		[49]
		*Sitophilus oryzae*		0.05	[50]
6	Caryophyllene (humulene)	*Blatella germanica*	N.A.		[47]
7	1,8-Cineole	*Pediculus humanus capitis*	77		[51]
		*Sitophilus oryzae*		0.084	[50]
8	Coniferyl alcohol	BxACE-1 from *Bursaphelenchus xylophilus*	1.06		[46]
		BxACE-2 from *Bursaphelenchus xylophilus*	1.41		[46]
		BxACE-3 from *Bursaphelenchus xylophilus*	1.13		[46]
9	Cymene	*Sitophilus oryzae*		0.05	[50]
		*Drosophila suzukii*	N.A.		[49]
10	Estragole (Allylanisole)	*Blatella germanica*	N.A.		[47]
11	Eugenol	*Sitophilus oryzae*		0.096	[50]
12	Isoeugenol	*Sitophilus oryzae*		0.11	[50]
13	Isosafrole	*Sitophilus oryzae*		0.71	[50]
14	Limonene	*Sitophilus oryzae*		0.73	[50]
		*Reticulitermes speratus* Kolbe	0.95		[41]
15	Linalool	*Sitophilus oryzae*	N.A.		[50]
16	Methyleugenol	*Sitophilus oryzae*		0.051	[50]
17	Menthol	*Sitophilus oryzae*		0.048	[50]
		*Drosophila suzukii*	N.A.		[49]
18	Menthone	*Sitophilus oryzae*		0.39	[50]
		*Drosophila suzukii*	N.A.		[49]
19	Nerolidol	BxACE-1 from *Bursaphelenchus xylophilus*	9.98		[46]
		BxACE-2 from *Bursaphelenchus xylophilus*	15.28		[46]
		BxACE-3 from *Bursaphelenchus xylophilus*	19.06		[46]
20	Nootkatone	*Musca domestica*	>30		[48]
		*Dermacentor variabilis*	>30		[48]
		*Periplaneta americana*	>30		[48]
		*Aedes aegypti*	>30		[48]
21	Ocimene	Japanese termite	0.96		[52]
		*Blatella germanica*	N.A.		[47]
22	Perilla aldehyde	*Drosophila suzukii*	3.06		[49]
23	Phellandrene	*Reticulitermes speratus* Kolbe	4.92		[41]
		*Blatella germanica*	2.2		[47]
24	α-Pinene	*Sitophilus oryzae*		0.44	[50]
		BxACE-1 from *Bursaphelenchus xylophilus*	0.24		[46]
		BxACE-2 from *Bursaphelenchus xylophilus*	0.64		[46]
		BxACE-3 from *Bursaphelenchus xylophilus*	0.68		[46]
		*Reticulitermes speratus* Kolbe	3		[41]
25	β-Pinene	BxACE-1 from *Bursaphelenchus xylophilus*	3.39		[46]
		BxACE-2 from *Bursaphelenchus xylophilus*	18.03		[46]
		BxACE-3 from *Bursaphelenchus xylophilus*	>50		[46]
		*Reticulitermes speratus* Kolbe	3.08		[41]
		*Sitophilus oryzae*		0.0028	[50]
26	α-Terpinene	*Sitophilus oryzae*		0.14	[50]
27	α-Terpineol	*Sitophilus oryzae*		3.94	[50]
28	β-Thujone	*Blatella germanica*	N.A.		[47]
29	Thymol	*Sitophilus oryzae*		0.57	[50]
		*Drosophila suzukii*	4.26		[49]

N.A.—the compound is not active or the inhibition is lower than 50%; IC_50_—Concentration of component that cause 50% inhibition of enzyme; Ki—inhibitory constant. BxACE-1, BxACE-2 and BxACE-3 are three different acetylcholinesterases found in *Bursaphelenchus xylophilus*. Values in mg/mL were recalculated by the authors of this paper.

**Table 2:** The EO components acting as the competitive and the noncompetitive inhibitors of AChE.

Competitive AChE Inhibitors	Reference	Noncompetitive AChE Inhibitors	Reference
Pulegon	[55]	Gossypol	[55]
Citral	[55]	Carvone	[60]
Linalool	[55]	Camphor	[60]
(−)-Bornyl acetate	[55]		
1,8-Cineol	[55,57,58]		
Terpinen-4-ol	[57]		
Fenchone	[60]		
γ-Terpinene	[60]		
Menthone	[50]		
Menthol	[50]		

**Table 3:** The intensification of the GABA-induced Cl^−^ current by EOs.

EO Components	Concentration of EOs Component (mM)	Change of GABA Current	Type of Receptor or Source of Receptor	Literature
(−)-Borneol	0.3	350%	α1β2γ2s GABA_A_	[76]
Camphor	0.3	40% (inhibition)	α1β2γ2s GABA_A_	[76]
Carvone	0.3	115%	α1β2γ2s GABA_A_	[76]
*cis*-Jasmone	1	250%	Bovine GABA_A_	[79]
Geraniol	1	500%	α1β1GABA_A_	[79]
(+)-Isomenthol	1	327%	α1β2γ2s GABA_A_	[78]
(+)-Isopulegol	0.3	380%	α1β2γ2s GABA_A_	[78]
Linalool	1	350%	α1β1GABA_A_	[79]
Nerolidol	1	150%	α1β1GABA_A_	[79]
Menthol	0.32	200%	α1β2γ2s GABA_A_	[78]
(+)-Menthol	0.1	596%	α1β2γ2s GABA_A_	[76]
(−)-Menthol	0.3	600%	α1β2γ2s GABA_A_	[76]
(−)-Menthone	0.3	150%	α1β2γ2s GABA_A_	[76]
Methyleugenol	0.03	280%	hippocampal neurons	[80]
Methyl jasmonate	1	230%	Bovine GABA_A_	[79]
α-Terpineol	1	299%	α1β2γ2s GABA_A_	[78]
α/β-Thujone	0.3	40% (inhibition)	α1β2γ2s GABA_A_	[76]
	0.1	715%	*Drosophila melanogaster* homomeric RDL_ac_ GABAr	[76]
α-Thujone	0.00066	208%	Rat GABA_A_	[81]
	0.003	70% (inhibition)	rat dorsal root ganglion neurons	[82]
Thymol	0.1	416%	α1β3γ2s GABA_A_	[75]
	0.01	150%	α1β1γ2s GABA_A_	[75]

Values in mg/mL were recalculated by the authors of this paper.

## Appendix A

**Table A1:** The effects of the essential oil components on the acetylcholinesterase activity in organisms other than insects.

No.	Essential Oil Components	AChE Source	IC_50_ (mM)	Ki (mM)	Reference
1	Anisaldehyde	Electric eel	N.A.		[122]
2	Anisole	Human erythrocyte	N.A.		[123]
3	Anethole	Electric eel	N.A.		[124]
		Electric eel			[67]
		Electric eel	0.88		[125]
		Electric eel	8.9		[126]
		Bovine erythrocyte	0.2		[127]
		Electric eel	N.A.		[122]
		Electric eel	N.A.		[128]
		Electric eel	0.87		[126]
4	Borneol	Bovine erythrocyte	N.A.		[64]
		Human erythrocyte	N.A.		[68]
		Bovine erythrocyte	N.A.		[63]
		Electric eel	N.A.		[122]
		Human erythrocyte	N.A.		[123]
		Electric eel	N.A.		[124]
5	Bornyl acetate	Electric eel		21.3	[55]
		Human erythrocyte	N.A.		[68]
		Bovine erythrocyte	N.A.		[63]
6	Camphene	Electric eel	N.A.		[122]
7	Camphor	Electric eel	0.05		[67]
		Electric eel	N.A		[124]
		Electric eel	11.2		[60]
		Human erythrocyte	N.A.		[68]
		Bovine erythrocyte	N.A.		[63]
		Electric eel	N.A.		[122]
		Human erythrocyte	N.A.		[123]
8	2-Carene	Bovine erythrocyte	0.9		[58]
9	3-Carene	Human erythrocyte	0.2		[68]
		Bovine erythrocyte	0.2		[58]
		Electric eel	0.26		[126]
10	Carvacrol	Electric eel	0.41		[65]
		Electric eel	0.61		[126]
		Electric eel	0.21		[122]
		Electric eel	0.76		[128]
11	Carvone	Electric eel	0.3		[67]
		Bovine erythrocyte	N.A.		[64]
		Electric eel	N.A.		[122]
		Electric eel	5.5		[60]
12	Caryophyllene (humulene)	Human erythrocyte	N.A.		[68]
		Bovine erythrocyte	0.13		[68]
		Electric eel	N.A.		[124]
		Human erythrocyte	N.A.		[68]
		Electric eel	N.A.		[129]
13	Caryophyllene oxide	Human erythrocyte	N.A.		[68]
		Bovine erythrocyte	N.A.		[63]
14	1,8-Cineole	Electric eel		0.025	[55]
		Electric eel	0.1		[124]
		Electric eel	0.71		[126]
		Bovine erythrocyte	0.26		[64]
		Electric eel	0.6		[51]
		Electric eel	0.84		[122]
		Human erythrocyte	0.4		[68]
		Electric eel	0.04	0.03	[57]
		Bovine erythrocyte	0.29	0.1	[58]
		Bovine erythrocyte	0.39		[63]
		Human erythrocyte	0.67		[130]
15	Cinnamaldehyde	Electric eel	N.A.		[122]
16	Cinnamyl alcohol	Electric eel	N.A.		[122]
17	Citral	Electric eel		7	[55]
		Electric eel	N.A.		[124]
		Electric eel	N.A.		[122]
18	Citronellal	Electric eel	N.A.		[122]
19	Citronellol	Electric eel	N.A.		[122]
20	Copaene	Human erythrocyte	N.A.		[68]
21	Cymene	Bovine erythrocyte	N.A.		[58]
22	Elemol	Bovine erythrocyte	0.16		[64]
23	Estragole (Allylanisole)	Electric eel	0.15		[67]
		Electric eel	12.6		[60]
		Electric eel	N.A.		[124]
		Electric eel	N.A.		[122]
24	Eugenol	Electric eel	2.9		[124]
		Electric eel	N.A.		[122]
		Human erythrocyte	N.A.		[123]
25	Fenchone	Electric eel	0.4		[67]
		Electric eel	7		[60]
26	Geraniol	Electric eel	0.1		[67]
		Electric eel	15		[60]
		Electric eel	N.A.		[122]
27	Globulol	Human erythrocyte	N.A.		[68]
28	Gossypol	Electric eel	1.5		[55]
29	Guaiol	Human erythrocyte	N.A.		[68]
30	Isoeugenol	Electric eel	N.A.		[122]
31	Limonene	Electric eel	N.A.		[124]
		Human erythrocyte	N.A.		[68]
		Electric eel	1.61		[125]
		Electric eel	4.33		[126]
		Electric eel	N.A.		[122]
		Bovine erythrocyte	N.A.		[64]
32	Linalool	Electric eel	0.3		[67]
		Electric eel		5.5	[55]
		Electric eel	N.A.		[124]
		Electric eel	15.6		[60]
		Electric eel	N.A.		[122]
		Human erythrocyte	N.A.		[68]
		Bovine erythrocyte	N.A.		[63]
		Bovine erythrocyte	N.A.		[64]
33	Linalyl acetate	Bovine erythrocyte	N.A.		[64]
		Electric eel	N.A.		[129]
34	Manool	Human erythrocyte	N.A.		[68]
35	Methylcinnamate	Electric eel	N.A.		[122]
36	Methyleugenol	Electric eel	N.A.		[122]
		Electric eel	N.A.		[124]
37	Menthofuran	Bovine erythrocyte	N.A.		[64]
38	Menthol	Bovine erythrocyte	N.A.		[64]
39	Menthone	Bovine erythrocyte	N.A.		[64]
		Electric eel	N.A.		[122]
40	Methol	Human erythrocyte	N.A.		[123]
41	Methoxycinnamaldehyde	Electric eel	N.A.		[124]
42	Methyl acetate	Bovine erythrocyte	N.A.		[64]
43	Myrcene	Electric eel	N.A.		[122]
44	Myrtenal	Electric eel	0.17		[122]
45	Nerol	Electric eel	N.A.		[122]
46	Nerolidol	Electric eel	N.A.		[122]
47	Neryl acetate	Human erythrocyte	N.A.		[68]
48	Phellandrene	Electric eel	0.88		[129]
49	Phenylethanol	Electric eel	N.A.		[122]
50	α-Pinene	Electric eel	0.16		[124]
		Electric eel	10.5		[130]
		Human erythrocyte	0.7		[68]
		Human erythrocyte	0.63		[131]
		Bovine erythrocytes	0.66		[63]
		Electric eel	N.A.		[122]
		Bovine erythrocytes	0.4		[58]
51	β-Pinene	Electric eel	N.A.		[124]
		Human erythrocyte	1.5		[68]
		Bovine erythrocyte	1.5		[63]
		Electric eel	N.A.		[122]
52	Piperitenone oxide	Bovine erythrocyte	0.38		[64]
53	Piperitenone	Bovine erythrocyte	0.72		[64]
		Bovine erythrocyte	0.83		[64]
54	Pulegone	Electric eel		0.85	[55]
		Bovine erythrocyte	0.89		[64]
55	Sabinene	Human erythrocyte	N.A.		[68]
		Electric eel	1.25		[125]
56	Sclareol	Human erythrocyte	N.A.		[68]
57	α-Terpinene	Bovine erythrocyte	N.A.		[58]
58	γ-Terpinene	Electric eel	0.2		[67]
		Electric eel	N.A.		[124]
		Electric eel	5.8		[60]
		Bovine erythrocyte	N.A.		[58]
59	α-Terpineol	Electric eel	8.43		[124]
		Human erythrocyte	N.A.		[68]
60	Terpinen-4-ol	Electric eel	20.7		[124]
		Electric eel	10.30	4.7	[57]
		Bovine erythrocyte	N.A.	2	[58]
		Electric eel	N.A.		[129]
		Electric eel	N.A.		[122]
61	Terpinolene	Electric eel	1.1		[129]
62	α-Thujone	Human erythrocyte	N.A.		[68]
		Electric eel	N.A.		[122]
63	Thymohydroquinone	Electric eel	0.24		[65]
64	Thymol	Electric eel	4.9		[65]
		Electric eel	1.39		[126]
		Electric eel	N.A.		[122]
65	Thymoquinone	Electric eel	0.85		[65]
66	Viridiflorol	Bovine erythrocyte	0.11		[64]
67	Verbenone	Electric eel	2.66		[122]
		Electric eel	0.73		[128]

N.A.—the compound is not active or the inhibition is lower than 50%; IC_50_—Concentration of component that cause 50% inhibition of enzyme; Ki—inhibitory constant. Values in mg/mL were recalculated by the authors of this paper.
